# Digital technology empowers exercise health management in older adults: a systematic review and meta-analysis of the effects of mHealth-based interventions on physical activity and body composition in older adults

**DOI:** 10.3389/fpubh.2025.1661028

**Published:** 2025-10-01

**Authors:** Guanbo Wang, Ranran Xiang, Xuemei Yang, Liang Tan

**Affiliations:** ^1^Physical Education Institute, Sichuan University of Science and Engineering, Zigong, China; ^2^School of Physical Education, Hunan University of Science and Technology, Xiangtan, China; ^3^Gdansk University of Physical Education and Sport, Gdansk, Poland

**Keywords:** digital technology, mobile health applications, older adults, physical activity, fitness

## Abstract

**Background:**

Prolonged Sedentary behavior (SB) and lack of Physical Activity (PA) in the older population significantly increase the risk of chronic disease development. The use of mobile health (mHealth) apps may have a positive impact on older adults, helping to increase their physical activity levels and optimize body composition. However, the effectiveness of mHealth-based interventions and potential moderators in this population is not fully understood.

**Objective:**

To assess the effectiveness of a mHealth-based intervention in promoting PA/moderate to vigorous physical activity (MVPA), reducing SB, and lowering body mass index (BMI) in older adults. The moderating effects of the mHealth intervention effects were also explored through subgroup analysis.

**Method:**

This study searched Embase, PubMed, Web of Science, and Cochrane databases (as of June 2025) to include randomized controlled trials (RCT) evaluating the effects of mHealth on PA, MVPA, SB, and BMI in older adults. Standardized mean differences (SMD) and 95% confidence intervals (CI) were calculated using random effects models.

**Results:**

A total of 14 RCTs were included (sample size = 2,511). mHealth intervention significantly elevated PA (SMD = 0.18, 95%CI: 0.01 to 0.35) and MVPA (SMD = 0.48, 95%CI: 0.20 to 0.75) and reduced SB (SMD = -0.55, 95% CI: −0.79 to −0.32), but no significant improvement in BMI (SMD = −0.27, 95% CI: −0.79 to 0.25). Subgroup analyses showed that: Commercial applications were better than research-based applications (PA: SMD = 0.18 vs. 0.07; MVPA: SMD = 0.70 vs. 0.31); more than 3 behavior change techniques (BCTs) interventions were effective for MVPA enhancement (SMD = 0.49) and SB reduction (SMD = −0.77); and the use of a theory paradigm intervention was more effective on SB reduction (SMD = −0.77 vs. 0.38).

**Conclusion:**

mHealth apps were effective in increasing PA/MVPA levels and reducing SB levels in older adults, but did not reach statistical significance in terms of BMI improvement. Through subgroup analyses, this study further found that commercial apps demonstrated greater strengths in promoting PA/MVPA; meanwhile, integrating more than 3 BCTs synergistically promoted MVPA levels and reduced SB.

## 1 Introduction

The global trend of population aging is increasing at an unprecedented rate. According to the latest report released by the United Nations, the global older population is expected to increase to 1.4 billion by 2030, and by 2050, the number will exceed 2.1 billion (1). Studies show that older adults are sedentary for an average of 9.4 h per day (2) and fail to meet current Physical Activity (PA) recommendations (3). The combination of prolonged sedentary behavior (Sedentary Behavior, SB) and low levels of PA results in a significant imbalance between PA and SB. This imbalance is accompanied by a significant increase in the prevalence of chronic non-communicable diseases (e.g., cardiovascular diseases, diabetes, osteoporosis, sarcopenia, and obesity) (4–11), which puts a tremendous strain on an individual’s quality of life, the burden of care on the family, and the social health-care system (12–14). Numerous research studies suggest that regular PA is the most cost-effective non-pharmacological intervention to improve health in old age, effectively reducing the risk of chronic disease, enhancing muscle function, and reducing the incidence of falls (15, 16). However, it is worrying that traditional health promotion programs (e.g., community fitness classes, paper-based promotional brochures) have significant limitations in terms of coverage, continuity, immediate feedback, and adherence management, making it difficult to provide personalized feedback (17, 18).

Currently, mobile health (mHealth) technologies, covering wearables, smartphones, tablets, mHealth apps, smartwatches, and pedometers, have gained widespread use in healthcare (19). Applying the mHealth app to exercise health management for older adults shows significant potential for enhancement: (1) provide personalized services across time and space constraints (20); (2) use sensing data (e.g., accelerometers) to enable immediate feedback and enhance self-efficacy (21); and (3) reach a wide range of people at low cost (22). However, there is a lack of systematic evaluation of the impact of stand-alone mHealth apps or apps that are part of coordinated interventions. In addition, research in this area has focused primarily on children, adolescents, and adults, with a notable lack of research in older populations. Although studies have examined the effectiveness of mHealth apps in promoting physical activity in different populations, there is significant heterogeneity in study design, intervention content, target populations, and outcome metrics, and findings vary (23–25). The study by Yerrakalva et al. (26), although it focused on older adults, was limited by the period, the limited amount of literature included, and the lack of inclusion of body composition as an outcome indicator, resulting in a lower overall quality of evidence.

In the field of research on mHealth app interventions, numerous theoretical frameworks, such as Self-Determination Theory (SDT), the Transtheoretical Model, the Health Belief Model, the Theory of Planned Behavior, and the Social Cognitive Theory (SCT), have been widely used to guide the design of relevant interventions (27). The number and type of Behavior Change Techniques (BCT) clusters are also seen as key factors influencing the effectiveness of mHealth app interventions (28). The standardized classification of BCT proposed by Migi et al. divides BCT into 16 clusters, including feedback and monitoring, rewards and threats, goals and planning, knowledge sharing, social support, and outcome comparison (29). Such categorization helps to clarify which BCT clusters are more effectively used in applications, which in turn promotes the promotion of PA and Physical Fitness (PF) (28). In summary, the effectiveness of mHealth app interventions is influenced by a variety of factors, including the type of app, intervention characteristics, theoretical paradigms, and BCT clusters. Despite the large number of reviews of mHealth app-based interventions in the existing literature, there are still significant gaps in the research.

The purpose of this study was to evaluate the effectiveness of a mHealth app-based intervention in promoting PA/moderate to vigorous physical activity (MVPA), reducing SB, and lowering body mass index (BMI) in older adults. Subgroup analyses were also conducted to explore the moderating effects of the mHealth intervention, such as the type of application, the theoretical framework, and the validity of the BCT clusters on the study’s findings. Provide targeted guidance recommendations for further advancing digital technology interventions for health promotion in older adult populations.

## 2 Methods

### 2.1 Registration and approval

This meta-analysis is reported following the PRISMA (Preferred Reporting Items for Systematic Reviews and Meta-Analyses) guideline (30). The current study was registered with the International Prospective Register of Systematic Reviews (PROSPERO), registration number CRD420251062721.

### 2.2 Search strategy

This study aimed to identify relevant randomized controlled trials (RCTs) published until June 2025 by searching Embase, PubMed, Web of Science, and Cochrane databases. The search strategy used a Boolean logic search method combining subject-related terms and free words. Search terms included: (aged, older adult), (mobile health apps* or mHealth apps* or portable software apps*), (physical activity or PA or MVPA or sedentary behavior), and (Physical Activity or PA or MVPA or Sedentary Behavior or SB or physical fitness) (See Supplementary Table S2). In addition, references cited in previously relevant review literature were reviewed in this study to identify relevant literature that may have been omitted.

### 2.3 Inclusion and exclusion criteria

#### 2.3.1 Inclusion criteria

The following inclusion criteria were adopted for this study: (1) Based on the World Health Organization’s definition of a healthy aging stem population, the subjects selected for the study were those aged 55 years and older (31), with no physical or cognitive dysfunction, and conditions such as overweight or obesity were not used as exclusion criteria. (2) Smartphone and tablet-based mHealth app interventions utilized in the study, which may include stand-alone apps or synergistic interventions. (3) The study design was an RCT. (4) The main outcome indicators included PA (mainly Steps), MVPA, SB, and BMI.

#### 2.3.2 Exclusion criteria

Exclusion criteria were as follows: (1) literature not in English; (2) duplicate publications, basic studies, observational studies, reviews, and case series-type articles; (3) studies that were not available in full text and had incomplete data; and (4) exclusion of mHealth apps that used text message-only interventions or that did not support smartphone or tablet operating systems.

### 2.4 Study selection

After the literature search, the initial search results were imported into EndNote 20 (Thomson ResearchSoft) to remove duplicate articles. Predefined inclusion and exclusion criteria were applied to the literature. Two researchers independently completed screening the titles and abstracts of the search results to identify literature that met the inclusion criteria, and further searches were conducted to obtain the full text of these documents. Finally, the full-text literature was screened.

### 2.5 Data extraction and management

Extracts included study characteristics (authors, year, country), participant characteristics (country, sample size, age, and BMI, among others), intervention characteristics (intervention, type of mHealth, theoretical paradigm, BCTa clusters, frequency and periodicity of the intervention), and outcome indicators (PA, MVPA, SB, and BMI). Missing data were resolved by consulting a third researcher when inconsistencies arose during the data extraction process.

### 2.6 Risk of bias and quality assessment

The quality of research in RCTs was systematically evaluated using the Cochrane Collaboration’s risk of bias assessment tool, which covers seven key areas: random sequence generation, allocation concealment, blinding of participants and personnel, blinding of outcome assessment, incomplete outcome data, selective reporting, and other bias (32). Each item was assessed into 3 levels: low, unclear, and high risk of bias. Each study was assessed as a whole based on the 7-item assessment, which was categorized into 3 levels: low risk of bias, unclear, and high risk of bias. Risk of bias figures will be generated by the software RevMan (Review Manager 5.3).

### 2.7 Statistical analysis

A meta-analysis of the included studies was conducted using a random effects model. For each outcome, we extracted the mean change (post-intervention minus pre-intervention) and standard deviation (SD) of the change in both the experimental group and the control group. However, if the mean and standard deviation were not reported, these results were calculated based on the standard error, median, range, and/or interquartile range (33, 34). In addition, the Get Data software is used to extract data from graphs when required. All data analyses were performed using Review Manager software version 5.3 or Stata software version 17.0, and standardized mean differences (SMDs) and 95% confidence intervals (CIs) were calculated according to the random-effects model, with *p* < 0.05 defined as a significant difference (35). Statistical heterogeneity between studies was examined using the Cochran Q test; we assessed publication bias by examining funnel plots. We also performed sensitivity analyses to test the robustness of the pooled results by excluding trials at risk of assessment bias.

## 3 Result

### 3.1 Study selection

**Figure 1:**
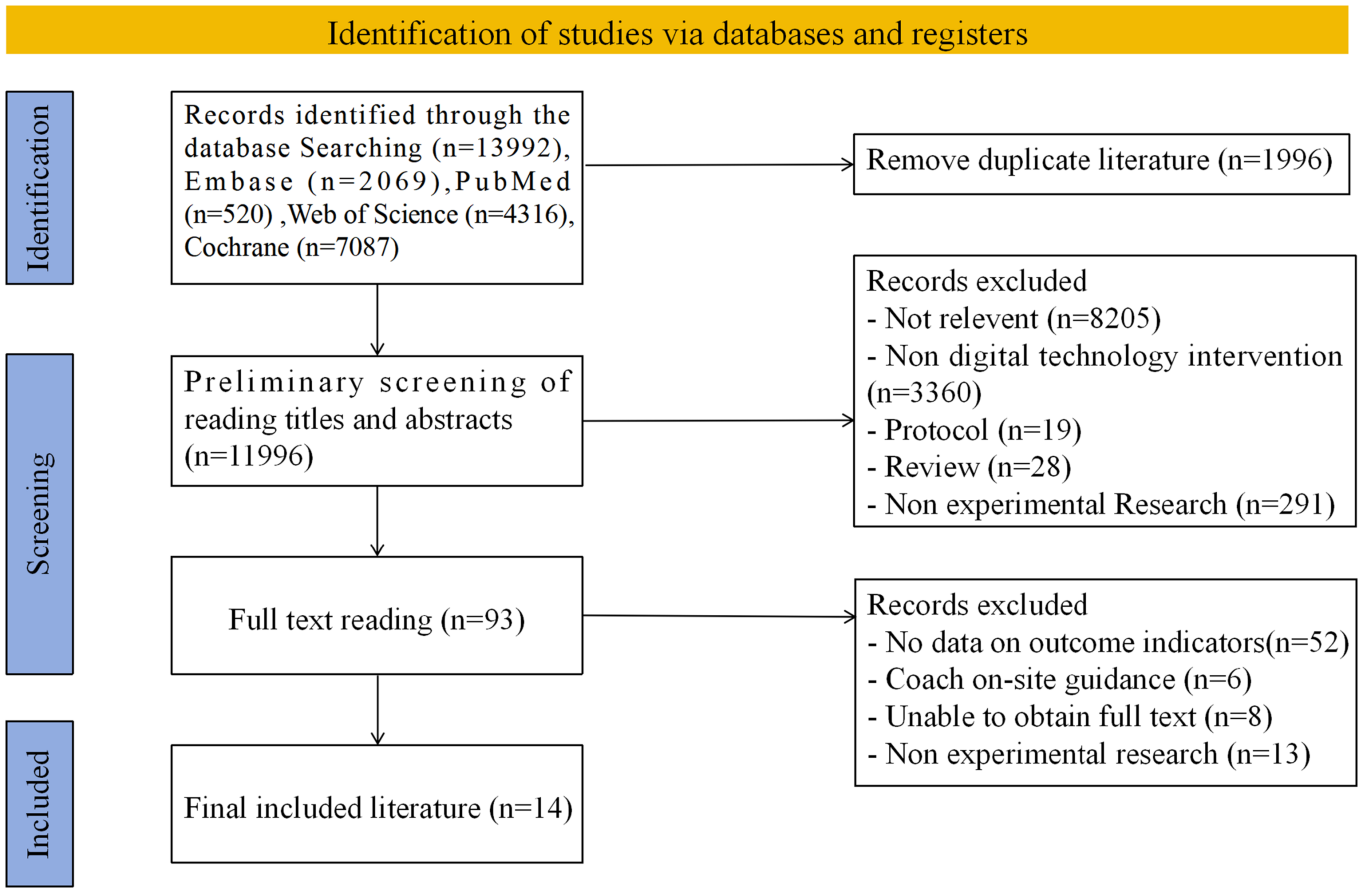
Flowchart of the study selection.

A total of 13,992 potentially relevant papers were identified after a comprehensive search of Embase (*n* = 2069), PubMed (*n* = 520), Web of Science (*n* = 4,316), and Cochrane (*n* = 7,087) databases. After removing duplicate references, 11,996 independent papers were finally screened. An initial review based on literature titles and abstracts was further narrowed down to 93 documents, which were subsequently reviewed in full text. Based on rigorous inclusion criteria, 14 kinds of literature were finally identified for inclusion in this research review (see Figure 1) (36–49).

### 3.2 Study characteristics

A total of 14 papers were included in this study, all of which were RCTs published between 2013 and 2025. The study sample size totaled 2,511 participants, with the number of participants in each study ranging from 8 to 254. As shown in Supplementary Table S3, the basic characteristics of the included studies were as follows: the age range of participants was 58 to 77 years. Eleven of the studies targeted overweight or obese populations. Of the 14 studies, 4 were conducted in North America, 4 in Asia, 3 in Oceania, and 3 in Europe. mHealth includes 9 commercial apps and 5 research apps. Mobile health apps are based on several theoretical paradigms, including Self-Regulation Theory (SRT), SDT, and SCT. Different numbers or types of BCT-based clusters were identified in the study, ranging from 3 to 6 clusters. Examples of BCT clusters applied include goal setting and planning, feedback, and monitoring. The intervention period ranged from 5 weeks to 24 months, with the most common training regimen being 3 times per week, and the frequency of interventions being predominantly 3 to 5 times per week, with a duration of approximately 30 to 35 min per session. The primary outcome indicators included PA (represented by steps), MVPA, SB, and BMI.

### 3.3 Risk of bias

**Figure 2:**
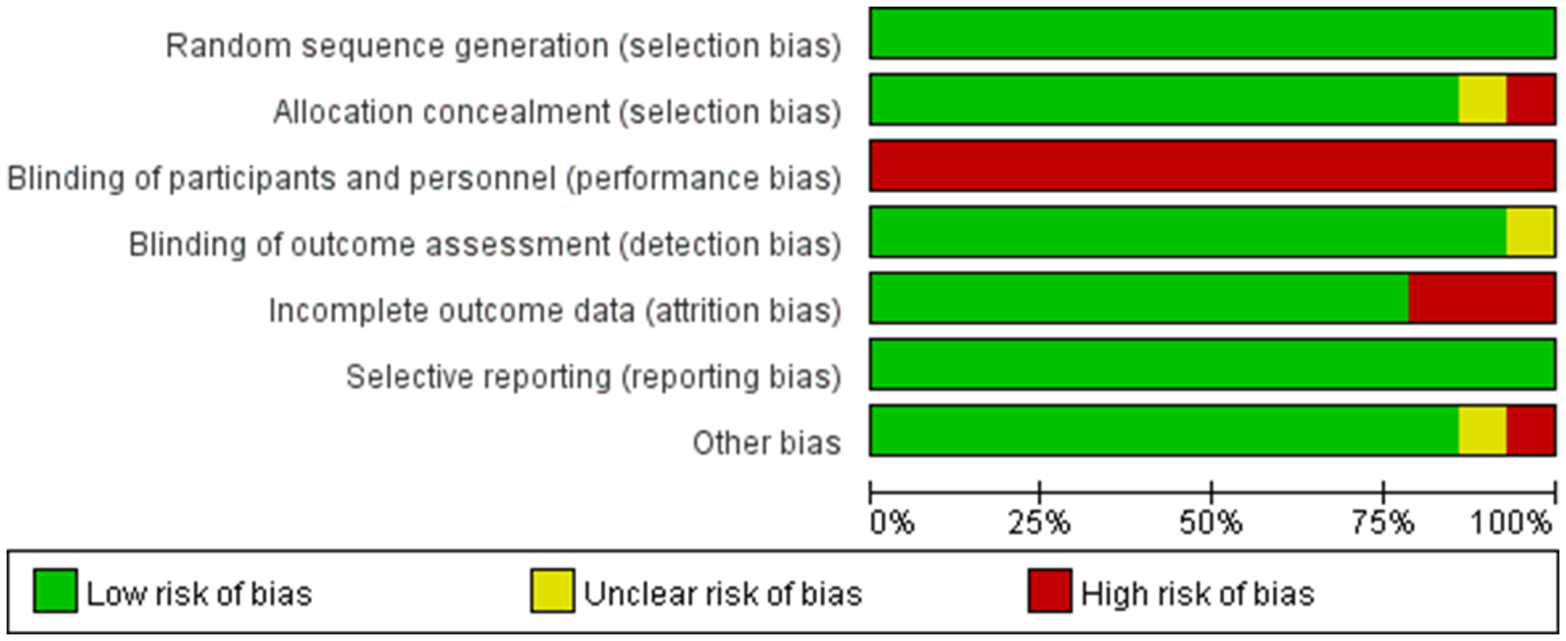
Summary of the risk of bias assessment.

**Figure 3:**
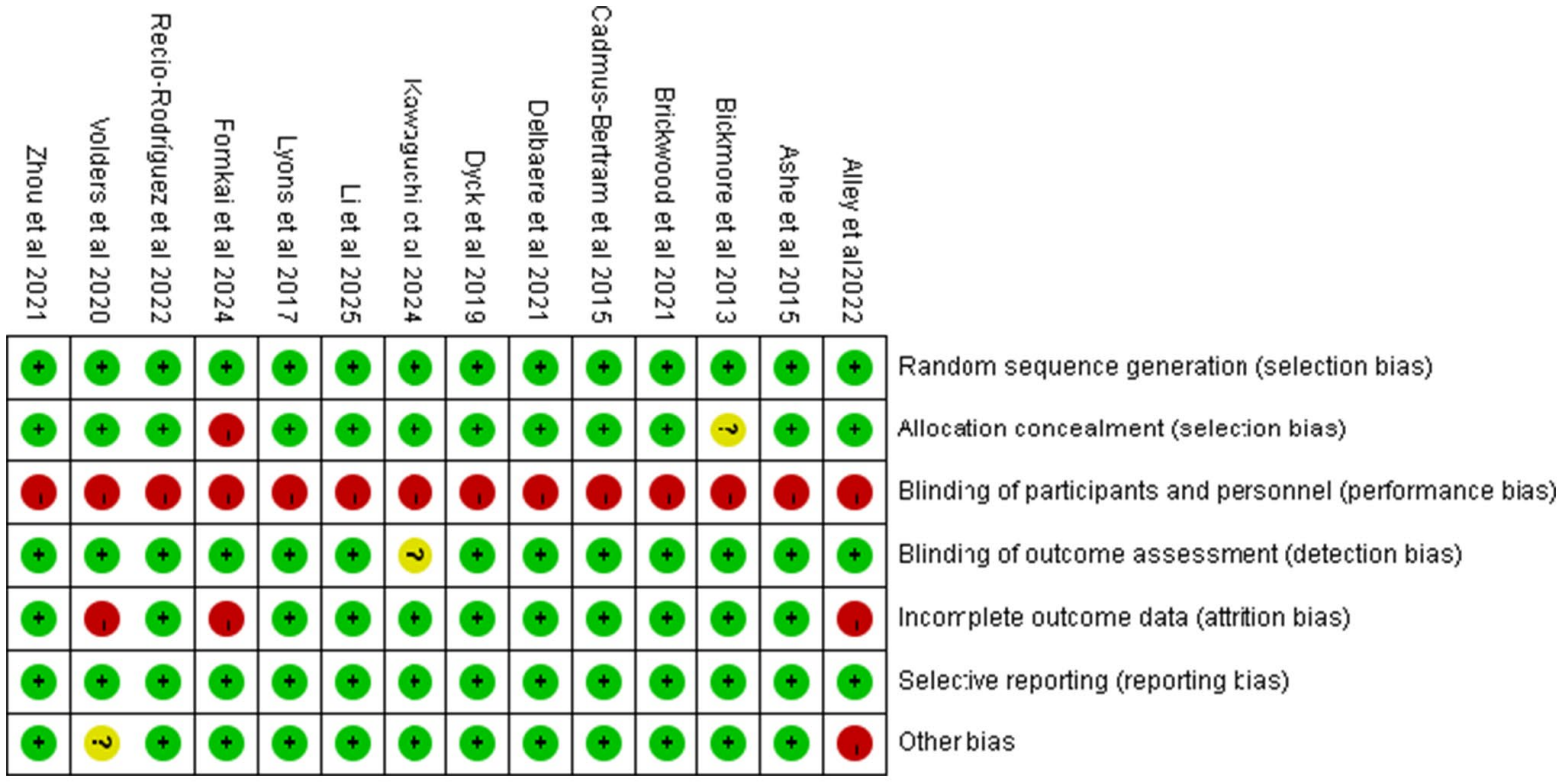
Risk of bias assessment results.

Based on the Cochrane Risk of Bias Assessment Tool, the 14 included studies were systematically evaluated for quality and risk of bias, and the results showed that 11 of them were at low risk of bias, while 3 studies were at high risk of bias. Figure 2 demonstrates the comprehensive results of the risk of bias assessment. Details of the risk of bias assessment for each study can be found in Figure 3.

### 3.4 Meta-analysis

#### 3.4.1 PA

**Figure 4:**
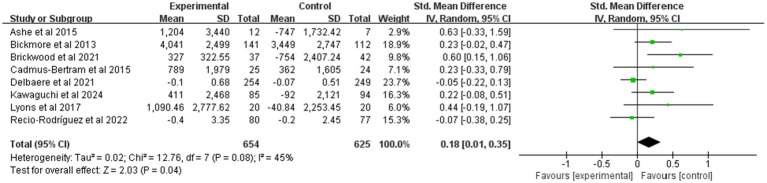
Forest plot of post-intervention PA value comparison between experimental and control groups. SD, standard deviation; Std, standardized; IV, inverse variance; CI, confidence interval.

Figure 4 summarizes the effects of mHealth on PA. This study used a random-effects model to make summary estimates of intervention effects. There were eight trials involving PA data from 1,279 participants. Overall, the mHealth intervention significantly elevated PA levels, with an effect size of 0.18 (95% CI: 0.01 to 0.35; *p* = 0.04; heterogeneity test *p* < 0.1).

#### 3.4.2 MVPA

**Figure 5:**
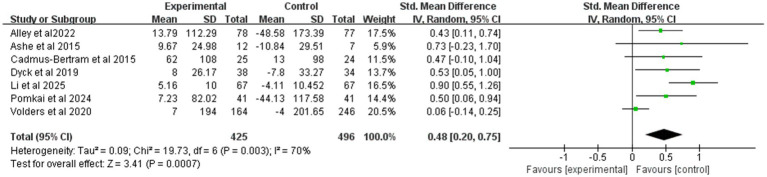
Forest plot of post-intervention MVPA value comparison between experimental and control groups. SD, standard deviation; Std, standardized; IV, inverse variance; CI, confidence interval.

Figure 5 synthesizes the effect of mHealth on MVPA. Pooled effects were estimated in this study using a random effects model. A total of 921 participants from seven trials provided MVPA data. Overall, the mHealth intervention significantly increased MVPA levels, with an effect size of 0.48 (95% CI: 0.2 to 0.75; *p* < 0.01; heterogeneity test *p* < 0.1).

#### 3.4.3 SB

**Figure 6:**
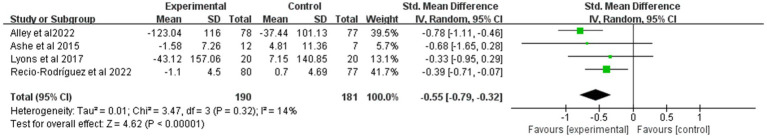
Forest plot of post-intervention SB value comparison between experimental and control groups. SD, standard deviation; Std, standardized; IV, inverse variance; CI, confidence interval.

Figure 6 presents the results of the pooled analysis of the effects of mHealth on SB. In this study, the pooled effects were estimated using a random effects model. A total of four trials involving 371 participants provided data on SB. Overall, exercise significantly reduced the level of sedentary behavior with an effect size of −0.55 (95% CI: −0.79 to −0.32; *p* < 0.01), and inter-study heterogeneity was not significant (*p* = 0.32).

#### 3.4.4 BMI

**Figure 7:**
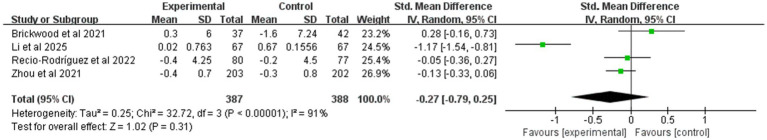
Forest plot of post-intervention BMI value comparison between experimental and control groups. SD, standard deviation; Std, standardized; IV, inverse variance; CI, confidence interval.

Figure 7 presents the results of the pooled analysis of the effect of mHealth on BMI. Pooled effects were estimated in this study using a random effects model. A total of 775 participants from 4 trials provided BMI data. Overall, the effect of exercise on reducing BMI levels was not significant, with an effect size of −0.27 (95% CI: −0.79 to 0.25; *p* = 0.31; heterogeneity test *p* < 0.1).

### 3.5 Subgroup analysis

#### 3.5.1 Subgroup analyses for PA

Subgroup analyses for different types of mHealth revealed that commercial mHealth (SMD = 0.18, *p* = 0.02) demonstrated a more significant effect in elevating PA levels compared to research mHealth (SMD = 0.07, *p* = 0.59). Whether or not a theoretical paradigm was used did not show statistical significance with different numbers of BCT interventions, implying that existing subgroup classifications are unable to elucidate effect size variance (Supplementary Figure S1).

#### 3.5.2 Subgroup analyses for MVPA

Subgroup analyses revealed that both mHealth interventions based on commercial (SMD = 0.70, *p* < 0.001) as well as interventions that included more than 3 BCTs (SMD = 0.49, *p* = 0.001) demonstrated significant effects. Of particular note, interventions based on the theoretical paradigm (SMD = 0.30, *p* = 0.02), as well as the non-reporting-theoretical-paradigm (NR) study group (SMD = 0.72, *p* < 0.001), showed significant differences. In contrast, interventions containing fewer than 3 BCTs failed to demonstrate statistical significance due to extreme heterogeneity (I^2^ = 89%) (Supplementary Figure S1).

#### 3.5.3 Subgroup analyses for SB

Subgroup analyses revealed that interventions based on theoretical paradigms (SMD = -0.77, *p* < 0.001) and interventions that included more than 3 BCTs (SMD = -0.77, *p* < 0.001) had significantly stronger effects compared to interventions in the NR cohort and with fewer than 3 BCTs (SMD = -0.38, *p* = 0.009). Notably, the results of the test for differences between subgroups showed borderline significance (*p* = 0.08) and there was an overlap of data between the Theoretical Paradigm group and the Multi-BCTs group, and between the NR group and the Fewer BCTs group, which implies that the number of BCTs may be a potential moderating variable contributing to differences in effect sizes (Supplementary Figure S1).

#### 3.5.4 Subgroup analyses for BMI

Subgroup analyses revealed no significant effects (*p* > 0.05) for all intervention groups. Although the *p*-value for the NR and multiple BCTs groups was 0.01, their 95% confidence intervals crossed the null line and still lacked statistical significance (Supplementary Figure S1).

### 3.6 Publication bias and sensitivity analysis

A visual interpretation of the funnel plot (Supplementary Figure S2) did not show signs of publication bias. A sensitivity analysis of the five groups of studies revealed the robustness of the overall results after excluding studies on an item-by-item basis (Supplementary Figure S3).

## 4 Discussion

This study comprehensively and integrally assessed the effect of mHealth interventions on PA, MVPA, SB, and BMI through a systematic review and meta-analysis. Results showed that mHealth-based interventions effectively elevated PA: (SMD = 0.18, 95%CI: 0.01 to 0.35) and MVPA (SMD = 0.48, 95%CI: 0.20 to 0.75) in older adults, while effectively reducing SB (SMD = −0.55, 95%CI: −0.79 to −0.32). However, its improvement in BMI did not reach statistical significance (SMD = −0.27, 95%CI: −0.79 to 0.25). Subgroup analyses showed significant variability in intervention effects: commercial-based applications were more effective than research-based applications in PA promotion (MVPA: SMD = 0.70 vs. 0.30), and strategies with more than 3 BCTs showed synergistic gains in the modulation of MVPA and SB (SMD = 0.49 vs. −0.77), whereas theoretical-paradigm groups showed a specific advantage only for SB reduction (SMD = −0.77). These findings confirm the core value of digital technology in empowering the health of the older adult, but also reveal the complexity and boundary conditions of the intervention effects.

In terms of facilitation mechanisms for PA and MVPA, this study found that commercial-based applications were significantly more effective than research-based applications. This phenomenon may stem from the fact that commercial products focus more on user experience design, e.g., enhancing motivation to participate through gamification elements (e.g., virtual medals, step leaderboards), whereas research applications tend to focus on functional completeness at the expense of interactive experience (23, 50). The results challenge the conventional wisdom that evidence-based design is necessarily superior to commercial products and suggest the critical role of practical design in the older population (23, 51). Of course, this could be attributed to the fact that mHealth, which is research-based, is still in the experimental stage, and its personalized design may not yet be fully developed (52).

The moderating effects of theoretical paradigms showed specificity (53). Interventions constructed based on SDT or SCT were particularly effective in reducing SB, possibly because theoretical frameworks are more adept at explaining intrinsically motivated-driven behavioral changes (e.g., autonomous choice of activity break rhythms) (54); However, their promotion of PA/MVPA was not consistent, suggesting that high-intensity activity may require more direct external incentives (e.g., stage-based rewards) (55). This finding provides an important rationale for the theoretical fit of future intervention designs.

Notably, the number of BCTs integrated significantly affected intervention efficacy: interventions that included more than three BCTs (e.g., goal-setting, real-time feedback, social support) showed a significant advantage in improving MVPA and reducing SB, confirming the “synergistic efficacy of BCTs” hypothesis proposed by Michie et al. (29). It has been shown that clusters of multiple BCTs can build self-regulating neural circuits and that goal setting can activate the dorsolateral prefrontal cortex (DLPFC) to enhance executive function and prospective memory (56, 57); Real-time feedback can enhance the computation of reward prediction errors and enhance the perception of reward for effort through basal nucleus dopaminergic projections (58); Social support, in turn, stimulates empathic motivation via the mirror neuron system (59). When these techniques cover the whole cycle of “goal formation-action monitoring-feedback regulation-strategy updating,” they can enhance the self-efficacy of older adults (56). Conversely, fragmented BCTs (≤3 BCTs) may result in a nonsignificant MVPA effect size due to failure to establish neurofunctional coupling (56).

It is thought-provoking that despite significant improvements in PA and SB, BMI did not show a synchronized decrease. This phenomenon may be attributed to the insufficient number of long-term intervention cycles included in the current study, resulting in a slow rate of BMI reduction. Improvements in PA can be achieved in a relatively short period, whereas reductions in BMI may take a longer time to show changes (60). Secondly, the increase in MVPA may not yet have reached the intensity threshold that has a significant impact on body fat metabolism. Therefore, future research should focus on two key areas: first, extending the intervention cycle to verify long-term effects (given that most existing research cycles are shorter than 12 weeks); second, adjusting changes in intervention intensity, especially mobile health (mHealth) interventions aimed at muscle strengthening (the proportion of such current studies is less than 10%) (61).

## 5 Strengths and limitations

In this study, a large amount of credible and relevant literature was collected by searching Embase, PubMed, Web of Science, and Cochrane databases. The PRISMA guidelines were strictly followed during the study, and a two-person independent screening process was used to minimize selection bias. The current study is the first systematic review and meta-analysis to comprehensively include RCTs to assess the effects of mHealth interventions on PA, MVPA, SB, and BMI in older adults. In subgroup analyses, this study is the first to explore the moderators of mHealth intervention effects, including the type of application, theoretical paradigm, and BCT clusters on various indicators in older adults. Therefore, this study is highly robust and innovative.

Inevitably, this study still has certain limitations. First, since some primary studies did not specify the exact names of the intervention apps, subgroup analyses could not be conducted within individual app types. Second, although the PRISMA guidelines were strictly followed, the limited number of primary studies means that certain subgroup-analysis results may still be heterogeneous. Finally, because the outcome measures used in the primary studies were not reported in identical units, these discrepancies could bias the findings and potentially limit the effect sizes.

## 6 Conclusion

This study revealed that the mHealth intervention had a significant effect in elevating PA and MVPA levels in older adults and was effective in reducing SB, but did not reach statistical significance in terms of BMI improvement. Through subgroup analyses, this study further found that commercial applications showed greater advantages in promoting PA/MVPA; Integration of more than three BCTs can synergistically contribute to increased MVPA levels and reduced SB. Based on these findings, it is recommended to prioritize the use of established commercial applications that incorporate multiple BCTs in health coaching practices. Future studies should incorporate trials with a longer period. Meanwhile, it is recommended that “intervention tools” and “measurement tools” be separated, and validated independent devices be used to evaluate the results so as to reduce bias.

## Data Availability

The original contributions presented in the study are included in the article/Supplementary material, further inquiries can be directed to the corresponding author.
