# Supplementary material for: Digital technology empowers exercise health management in older adults: a systematic review and meta-analysis of the effects of mHealth-based interventions on physical activity and body composition in older adults
Source: Front Public Health. 2025 Oct 1;13:1661028. doi: 10.3389/fpubh.2025.1661028 (PMC12521191; doi:10.3389/fpubh.2025.1661028)
Supplement: Supplementary file 1 [file Table_1.DOCX]

# Appurtenant material

[Table S1. PRISMA meta-analysis checklist 2](#_Toc7198)

[Table S2. Search strategy（Embase） 5](#_Toc11952)

[Table S3. Characteristics of included studies 6](#_Toc606)

Figure S1. Subgroup analysis. 8

Figure S2. Assessment of potential publication bias by funnel plots. 11

Figure S3. Sensitivity analysis. 13

#### Table S1. PRISMA meta-analysis checklist

| **Section and Topic** | **Item #** | **Checklist item** | **Location where item is reported** |
| --- | --- | --- | --- |
| **TITLE** | | |  |
| Title | 1 | Identify the report as a systematic review. | 1 |
| **ABSTRACT** | | |  |
| Abstract | 2 | See the PRISMA 2020 for Abstracts checklist. | 1 |
| **INTRODUCTION** | | |  |
| Rationale | 3 | Describe the rationale for the review in the context of existing knowledge. | 2 |
| Objectives | 4 | Provide an explicit statement of the objective(s) or question(s) the review addresses. | 2 |
| **METHODS** | | |  |
| Eligibility criteria | 5 | Specify the inclusion and exclusion criteria for the review and how studies were grouped for the syntheses. | 4 |
| Information sources | 6 | Specify all databases, registers, websites, organisations, reference lists and other sources searched or consulted to identify studies. Specify the date when each source was last searched or consulted. | 4 |
| Search strategy | 7 | Present the full search strategies for all databases, registers and websites, including any filters and limits used. | Table S2 |
| Selection process | 8 | Specify the methods used to decide whether a study met the inclusion criteria of the review, including how many reviewers screened each record and each report retrieved, whether they worked independently, and if applicable, details of automation tools used in the process. | 4 |
| Data collection process | 9 | Specify the methods used to collect data from reports, including how many reviewers collected data from each report, whether they worked independently, any processes for obtaining or confirming data from study investigators, and if applicable, details of automation tools used in the process. | 5-6 |
| Data items | 10a | List and define all outcomes for which data were sought. Specify whether all results that were compatible with each outcome domain in each study were sought (e.g. for all measures, time points, analyses), and if not, the methods used to decide which results to collect. | Table S3 |
|  | 10b | List and define all other variables for which data were sought (e.g. participant and intervention characteristics, funding sources). Describe any assumptions made about any missing or unclear information. | Table S3 |
| Study risk of bias assessment | 11 | Specify the methods used to assess risk of bias in the included studies, including details of the tool(s) used, how many reviewers assessed each study and whether they worked independently, and if applicable, details of automation tools used in the process. | Figure S2 |
| Effect measures | 12 | Specify for each outcome the effect measure(s) (e.g. risk ratio, mean difference) used in the synthesis or presentation of results. | 5-6 |
| Synthesis methods | 13a | Describe the processes used to decide which studies were eligible for each synthesis (e.g. tabulating the study intervention characteristics and comparing against the planned groups for each synthesis (item #5)). | 5-6 |
|  | 13b | Describe any methods required to prepare the data for presentation or synthesis, such as handling of missing summary statistics, or data conversions. | 5-6 |
|  | 13c | Describe any methods used to tabulate or visually display results of individual studies and syntheses. | 5-6 |
|  | 13d | Describe any methods used to synthesize results and provide a rationale for the choice(s). If meta-analysis was performed, describe the model(s), method(s) to identify the presence and extent of statistical heterogeneity, and software package(s) used. | 5-6 |
|  | 13e | Describe any methods used to explore possible causes of heterogeneity among study results (e.g. subgroup analysis, meta-regression). | 5-6 |
|  | 13f | Describe any sensitivity analyses conducted to assess robustness of the synthesized results. | 5-6 |
| Reporting bias assessment | 14 | Describe any methods used to assess risk of bias due to missing results in a synthesis (arising from reporting biases). | 5-6 |
| Certainty assessment | 15 | Describe any methods used to assess certainty (or confidence) in the body of evidence for an outcome. | Figure S2 |
| **RESULTS** | | |  |
| Study selection | 16a | Describe the results of the search and selection process, from the number of records identified in the search to the number of studies included in the review, ideally using a flow diagram. | Figure 1 |
|  | 16b | Cite studies that might appear to meet the inclusion criteria, but which were excluded, and explain why they were excluded. | Figure 1 |
| Study characteristics | 17 | Cite each included study and present its characteristics. | 7 |
| Risk of bias in studies | 18 | Present assessments of risk of bias for each included study. | 8 |
| Results of individual studies | 19 | For all outcomes, present, for each study: (a) summary statistics for each group (where appropriate) and (b) an effect estimate and its precision (e.g. confidence/credible interval), ideally using structured tables or plots. | 6-12 |
| Results of syntheses | 20a | For each synthesis, briefly summarise the characteristics and risk of bias among contributing studies. | 12 |
|  | 20b | Present results of all statistical syntheses conducted. If meta-analysis was done, present for each the summary estimate and its precision (e.g. confidence/credible interval) and measures of statistical heterogeneity. If comparing groups, describe the direction of the effect. | 6-12 |
|  | 20c | Present results of all investigations of possible causes of heterogeneity among study results. | 12 |
|  | 20d | Present results of all sensitivity analyses conducted to assess the robustness of the synthesized results. | 12 |
| Reporting biases | 21 | Present assessments of risk of bias due to missing results (arising from reporting biases) for each synthesis assessed. | 12 |
| Certainty of evidence | 22 | Present assessments of certainty (or confidence) in the body of evidence for each outcome assessed. | 12 |
| **DISCUSSION** | | |  |
| Discussion | 23a | Provide a general interpretation of the results in the context of other evidence. | 12-13 |
|  | 23b | Discuss any limitations of the evidence included in the review. | 13 |
|  | 23c | Discuss any limitations of the review processes used. | 13 |
|  | 23d | Discuss implications of the results for practice, policy, and future research. | 13 |
| **OTHER INFORMATION** | | |  |
| Registration and protocol | 24a | Provide registration information for the review, including register name and registration number, or state that the review was not registered. | 4 |
|  | 24b | Indicate where the review protocol can be accessed, or state that a protocol was not prepared. | Not Applicable |
|  | 24c | Describe and explain any amendments to information provided at registration or in the protocol. | Not Applicable |
| Support | 25 | Describe sources of financial or non-financial support for the review, and the role of the funders or sponsors in the review. | 15 |
| Competing interests | 26 | Declare any competing interests of review authors. | 15 |
| Availability of data, code and other materials | 27 | Report which of the following are publicly available and where they can be found: template data collection forms; data extracted from included studies; data used for all analyses; analytic code; any other materials used in the review. | Not Applicable |

*From:*  Page MJ, McKenzie JE, Bossuyt PM, Boutron I, Hoffmann TC, Mulrow CD, et al. The PRISMA 2020 statement: an updated guideline for reporting systematic reviews. BMJ 2021;372:n71. doi: 10.1136/bmj.n71

For more information, visit: <http://www.prisma-statement.org/>

#### Table S2. Search strategy（Embase）

| No. Query Results Results Date  #5. #1 AND #2 AND #3 AND #4 2,069 2 Jun 2025  #4. 'randomized controlled trial':ab,ti OR 1,442,863 2 Jun 2025  'randomized':ab,ti OR 'placebo':ab,ti  #3. 'sedentar*':ab,ti OR 'sitting':ab,ti OR 10,007,418 2 Jun 2025  'inactiv*':ab,ti OR 'activ*':ab,ti OR  'walk*':ab,ti OR 'strength*':ab,ti OR  'exercise':ab,ti OR 'mvpa':ab,ti OR 'steps':ab,ti  OR 'step':ab,ti OR 'fit':ab,ti OR 'fitness':ab,ti  OR 'vo2':ab,ti OR 'maximal oxygen uptake':ab,ti  #2. 'app':ab,ti OR 'apps':ab,ti OR 'app-based':ab,ti 140,142 2 Jun 2025  OR 'mhealth':ab,ti OR 'm-health':ab,ti OR  'smartphone*':ab,ti OR 'smart phone*':ab,ti OR  'mobile phone*':ab,ti OR 'cell phone*':ab,ti OR  'cellphone*':ab,ti OR 'e-health':ab,ti OR  'ehealth':ab,ti  #1. 'elder*':ab,ti OR 'old*':ab,ti OR 'senior*':ab,ti 426,066 2 Jun 2025  OR 'adult':ab,ti OR 'veteran*':ab,ti OR  'geriatric*':ab,ti OR 'retire*':ab,ti OR 'aged':ab,ti |
| --- |

#### Table S3. Characteristics of included studies

| **Study** | **Country** | **Characteristics of subject** | | | | **Interventions information** | | | | | |
| --- | --- | --- | --- | --- | --- | --- | --- | --- | --- | --- | --- |
|  |  | Number | Age (mean[SD]) | BMI (mean[SD]) | Participants characteristics | Interventions measure | Type of mHealth | Theoretical paradigm | BCTa clusters | Intervention frequency/period | Outcome |
| Ashe et al  2015^1^ | Canada | 12/8 | 64.8 ± 4.6 | 26.9 ± 6.8 | overweight | Group education, individualized PA prescription, Fitbit with the Fitbit app use | Commercial | Social Ecological Model、SCT | Group Education & Social Support、Individualized Prescription、Self-Monitoring、Goal Setting、Environmental Cues、Behavior Substitution | 4 time/ weekly, 6 months | PA、MVPA、SB |
| Alley et al 2022^2^ | Australia | 78/77 | 69.88 ± 4.10 | 29.34 ± 28.40 | overweight | Web-based tailored advice + Fitbit Activity track | Commercial | Theory of Planned Behavior、SCT | Self-Monitoring、Goal Setting、Feedback & Reinforcement、Computer-Tailored Advice、Barrier Identification & Problem Solving、Social Support | 5 times/week, 12 weeks | MVPA、SB |
| Bickmore et al 2013^3^ | USA | 114/112 | 71.7 ± 5.6 | 29.6 | overweight | Tablet with an embodied conversational agent. app and pedometer | Research | SCT、Embodied Conversational Agent Theory | Conversational Interaction、Goal Negotiation、Feedback、Problem Solving、Self-Efficacy Enhancement | Instructed to have 1 conversation with ECA per day,2 months | PA |
| Brickwood et al 2021^4^ | Australia | 37/42 | 72.3 ±7 | 30.2 ±6.1 | obese or overweight | Jawbone UP24 (TM; Jawbone, Inc) AT and ZTE (TM) mobile device and data plan. | Commercial | SCT、SDT | Text Message Feedback、Dynamic Goal Adjustment、Self-Monitoring、Telephone Counseling、Social Support | 30min/day, 5 times/week, 12 weeks | PA、BMI |
| Cadmus-Bertram et al 2015^5^ | USA | 25/24 | 58.6 ± 6.5 | 29.2 ±3.8 | obese or overweight | ActiGraph GT3X+Web-based tailored advice + Fitbit Activity track | Commercial | SRT, CALO-RE framework. | Self-Monitoring、Goal Setting、Knowledge Education、Action Planning、Feedback | 150min/week, 16 week | PA、MVPA |
| Delbaere et al 2021^6^ | Australia | 254/249 | 77.1 ± 5.5 | 27.3 ±4.5 | overweight | Group education,iPad and app/McRoberts MoveMonitor Wearable device. Initial visit then telephone counseling | Research | SCT | Balance & Gait Training、Goal Setting、Self-Monitoring、Feedback、Environmental Cues |  | PA |
| Dyck et al 2019^7^ | Belgium | 38/34 | 70.8 ± 4.1 | 26.0 ± 4.2 | overweight | MyPlan2.0, ActiGraph GT3X+ accelerometer. processed using Actilife 6.13.3 | Research | SRT | Action Planning、Coping Planning、Goal Adaptation、Self-Monitoring、Feedback | 1time /week, 5 week | MVPA |
| Kawaguchi et al 2024^8^ | Japan | 85/94 | 69.9 ± 6 |  |  | ESP app | Commercial | NR | Self-Monitoring、Goal Setting、Feedback | 4time/week,12 weeks | PA |
| Li et al2025^9^ | China | 67/67 | 69.3 ± 5.1 | 24.42 ± 3.15 |  | Smartphone App (synchronizable with wearable device ActiGraph wGT3X-BT) and Web portal | Commercial | NR | Incentive Mechanisms、Self-Monitoring、Social Support | 30min/day, 5 times/week, 6 months | PA、BMI |
| Lyons et al  2017^10^ | USA | 20/20 | 61.25 ± 5.00 | 30.00 ± 2.86 | obese or overweight | iPad and app/UP24 Jawbone Wearable device. Initial visit then telephone counseling | Commercial | NR | Self-Monitoring、Goal Setting、Feedback | Weekly telephone counseling,3 months | PA、SB |
| Pomkai et al 2024^11^ | Thailand | 41/41 | 63.9 ± 9.0 | 25.2 ± 3.4 | overweight | LINE application and ActiGraph wGT3X-BT accelerometer | Research | NR | Personalized Intervention、Goal Setting & Tracking、Feedback | 30-45 min/day, 7 times/week, 8 weeks | MVPA |
| Recio‑Rodríguez et al 2022^12^ | Spain | 80/77 | 69.9 ± 3.6 | 28.2 ± 4.2 | overweight | Smartphone wristband + personalized APP | Commercial | NR | Self-Monitoring、Feedback、Goal Setting | 150min/week,3 months | PA、BMI、SB |
| Volders et al 2020^13^ | Netherlands | 164/246 | 74.20 ± 6.60 | 26.9 ± 5.42 | overweight | Computer Customization Recommendation Letter +Online Platform  +ActiGraph GT3X-BT | Research | SCT、Self-Efficacy Theory | Goal Setting、Self-Efficacy Enhancement、Feedback | 3 time/4 months | MVPA |
| Zhou et al 2021^14^ | China | 203/202 | 69.7 ± 5.3 | 27.6 ± 2.9 | overweight | Smart wristband (for recording steps and activity intensity) and Bluetooth scale, which automatically synchronize data to the cloud and generate charts for feedback. | Commercial | NR | Self-Monitoring、Goal Setting、Feedback | 3 time /week,3 months | BMI |

PA: physical activity; SB: sedentary behavior; MVPA: moderate to vigorous physical activity; BMI: Body Mass Index; BCT: behavior change technique; SRT: self-regulation theory; SDT: self-determination theory; SCT: social cognitive theory; NR: not reported.

#### Figure S1. Subgroup analysis. (a) PA. (b) MVPA. (c) SB. (d) BMI.

.
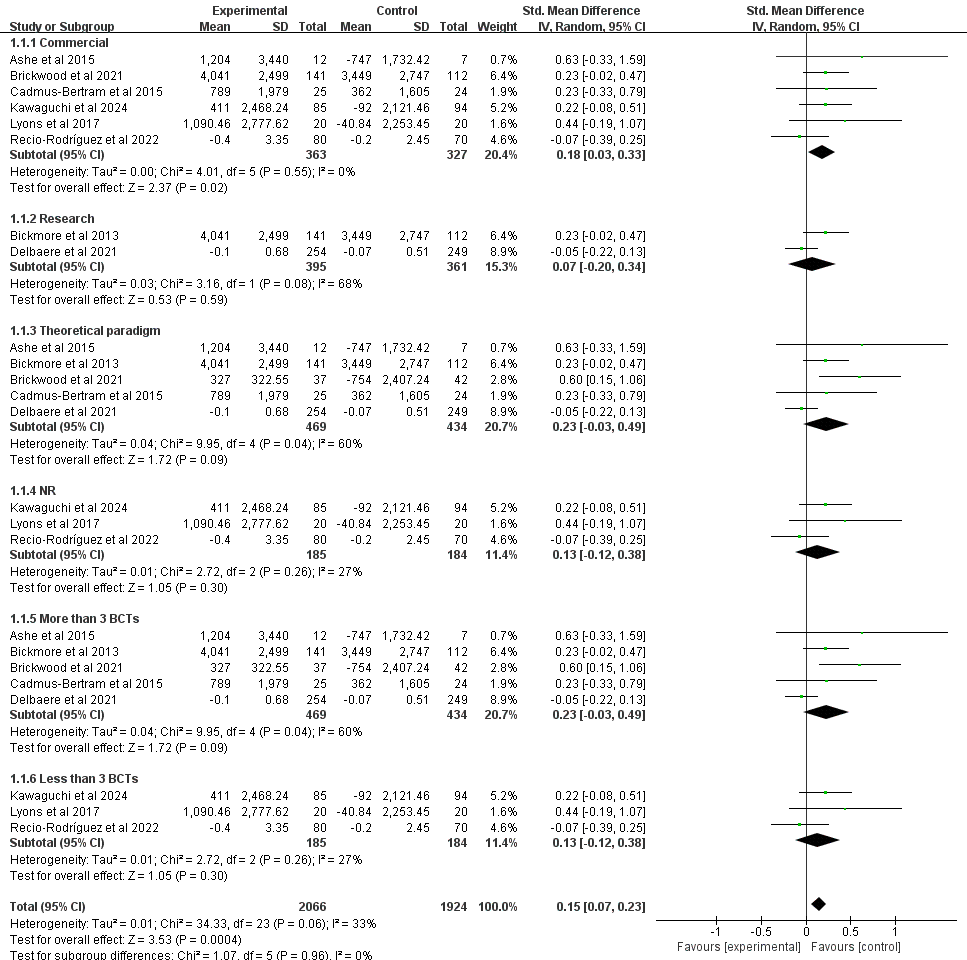


(a) PA.


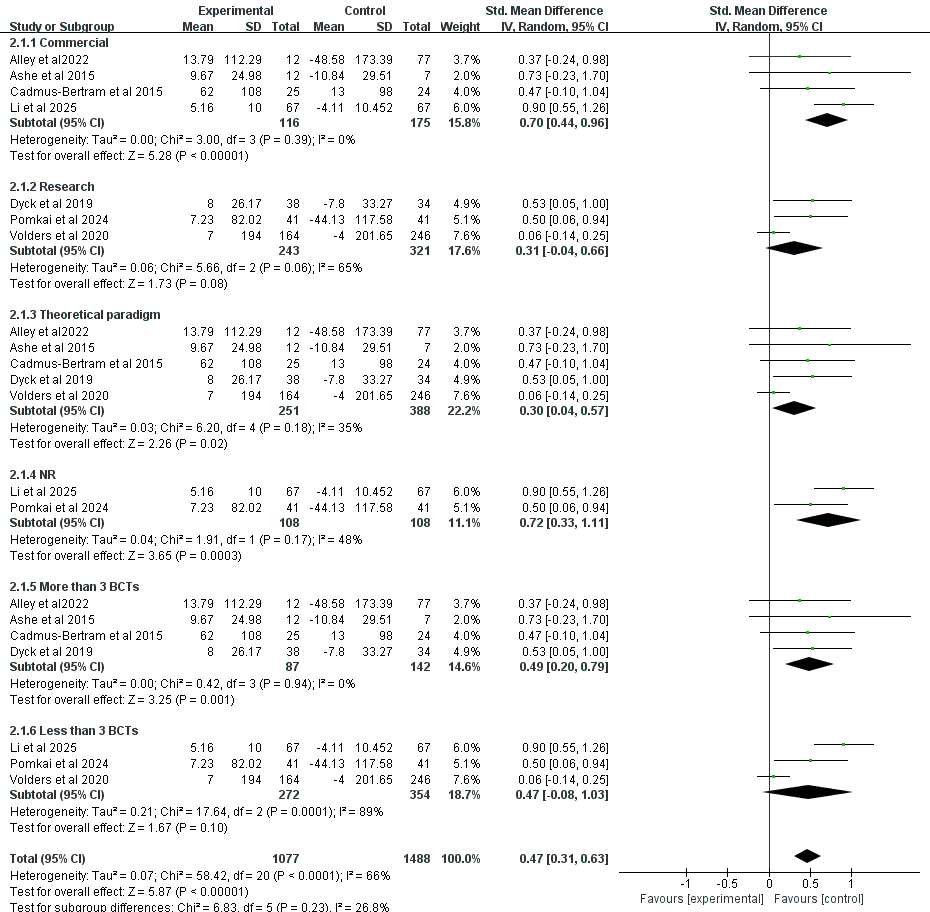


(b) MVPA.


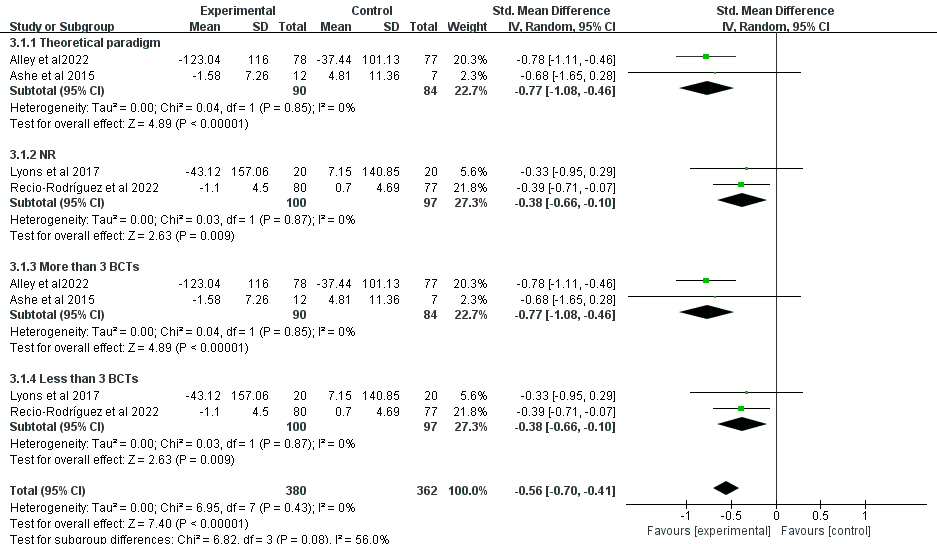


(c) SB.


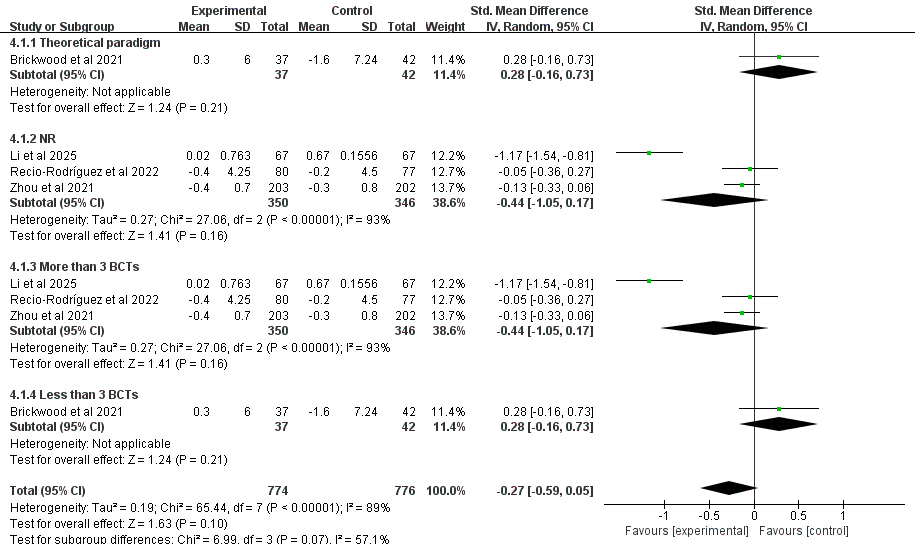


(d)BMI.

#### Figure S2. Assessment of potential publication bias by funnel plots. (a) PA. (b) MVPA. (c) SB. (d)BMI.

**
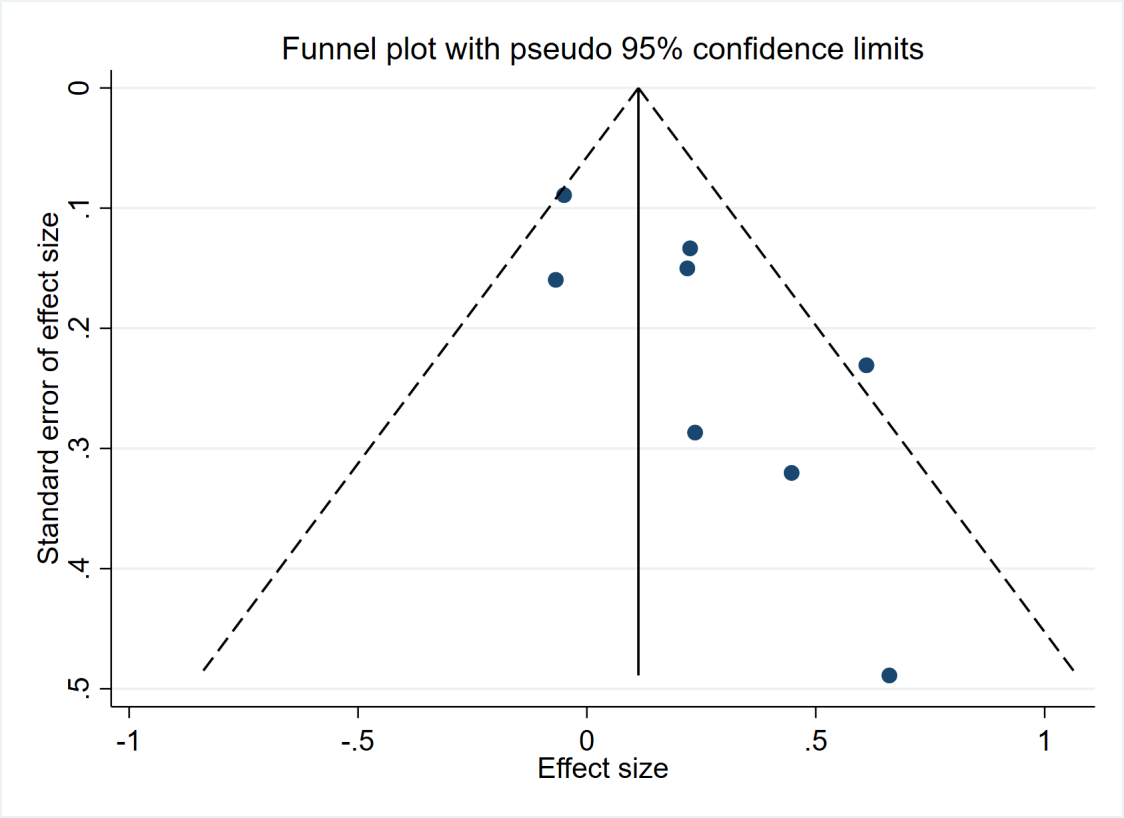
**

(a) PA.

**
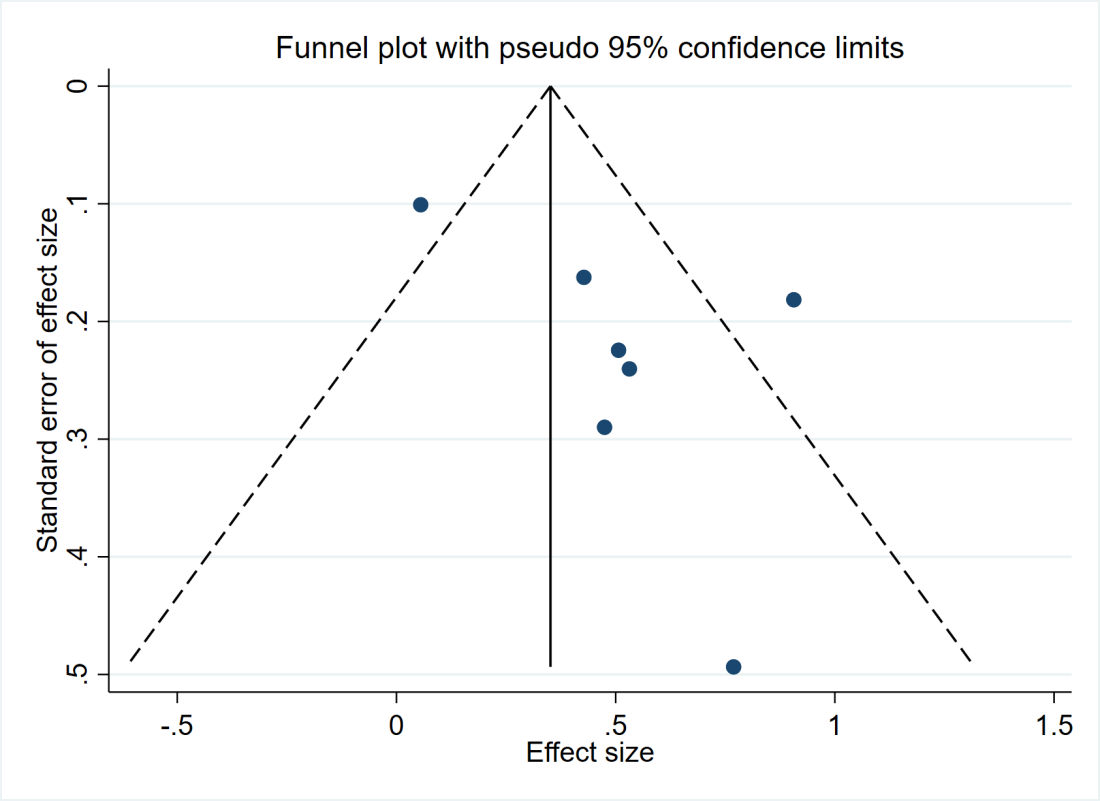
**

(b) MVPA.

**
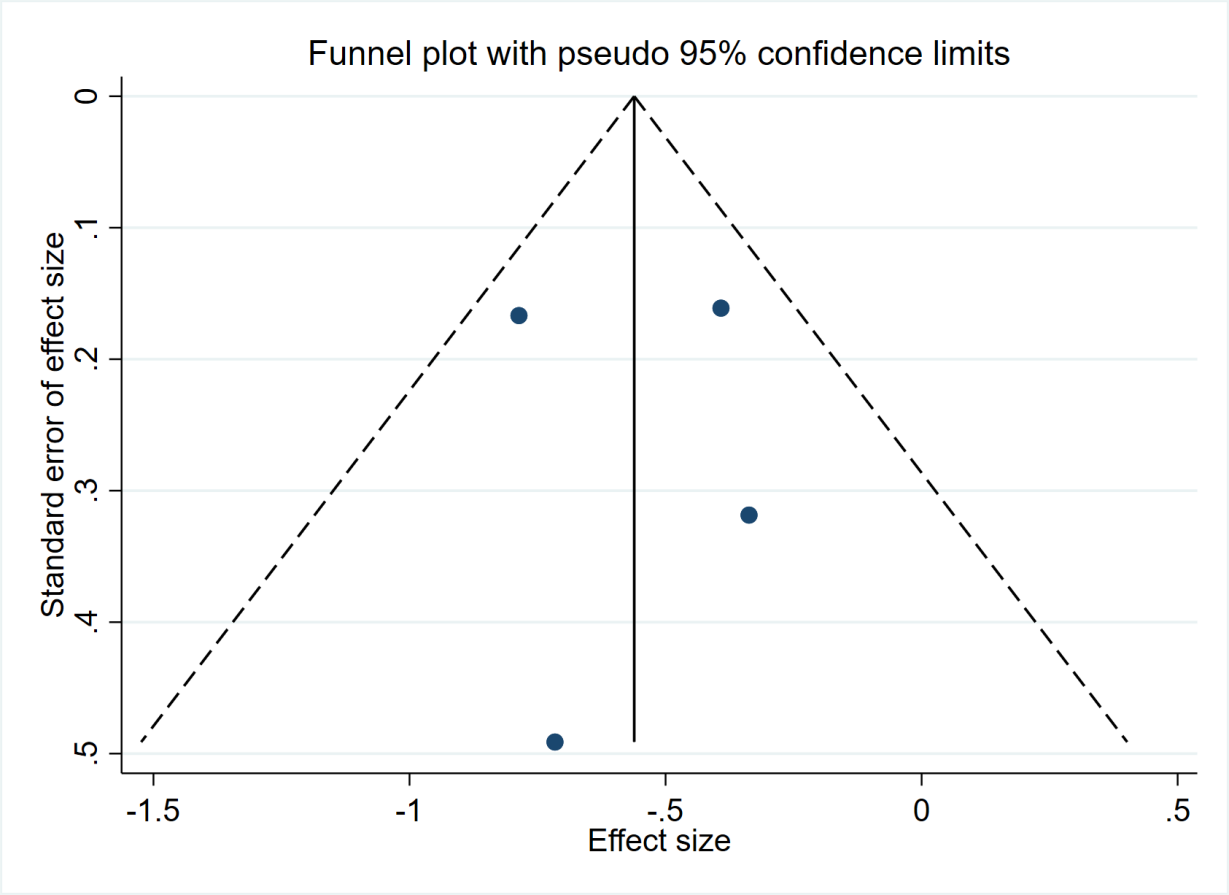
**

(c) SB.

**
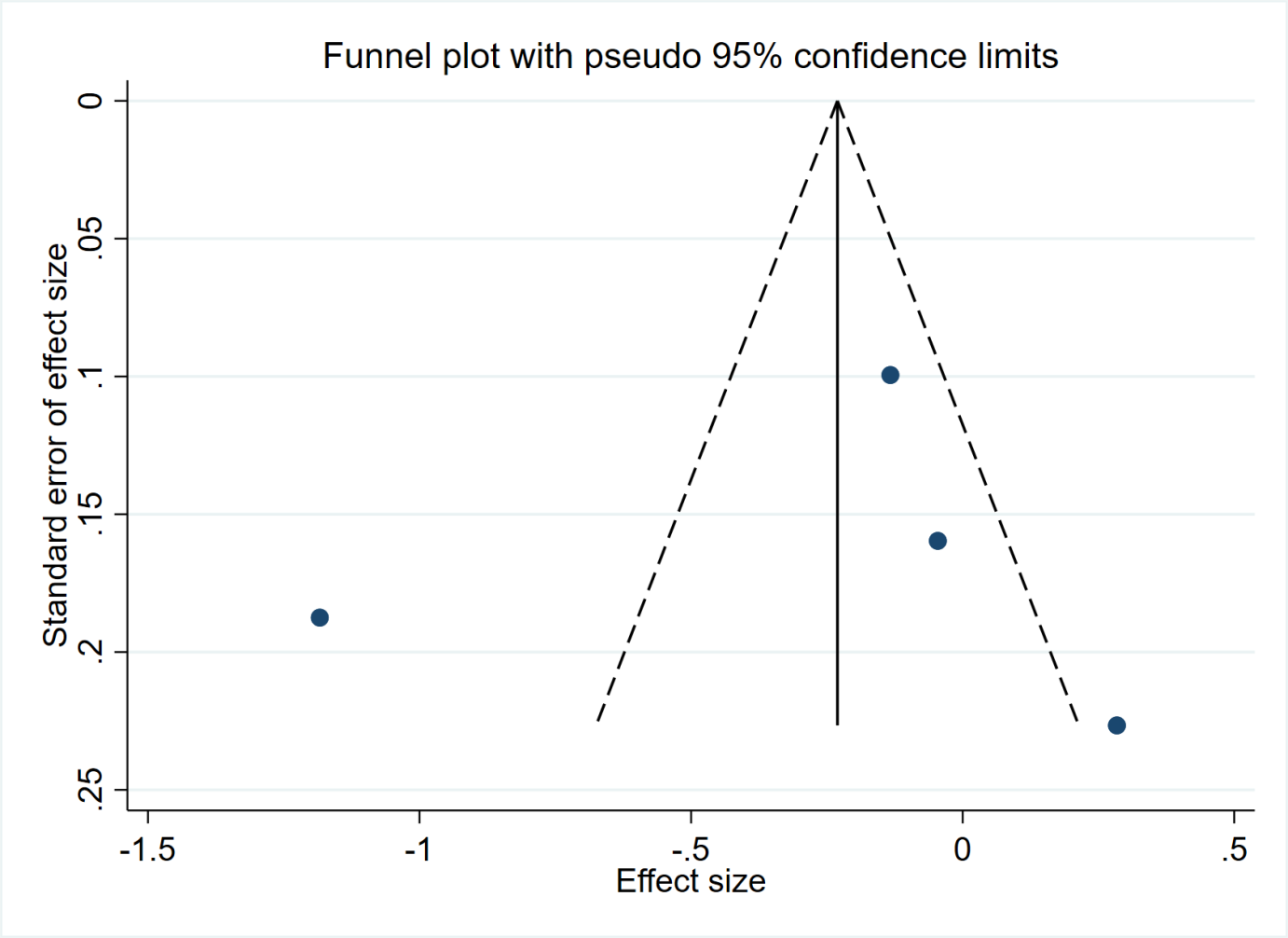
**

(d) BMI.

#### Figure S3. Sensitivity analysis. (a) PA. (b) MVPA. (c) SB. (d) BMI.

(a) PA.

(b) MVPA.

(c) SB

(d) BMI

1. Ashe MC, Winters M, Hoppmann CA, et al. "Not just another walking program": Everyday Activity Supports You (EASY) model-a randomized pilot study for a parallel randomized controlled trial. *Pilot Feasibility Stud.* 2015;1: 4.

2. Alley SJ, van Uffelen J, Schoeppe S, et al. The Effectiveness of a Computer-Tailored Web-Based Physical Activity Intervention Using Fitbit Activity Trackers in Older Adults (Active for Life): Randomized Controlled Trial. *Journal of Medical Internet Research.* 2022;24(5).

3. Bickmore TW, Silliman RA, Nelson K, et al. A Randomized Controlled Trial of an Automated Exercise Coach for Older Adults. *Journal of the American Geriatrics Society.* 2013;61(10): 1676-1683.

4. Brickwood K-J, Ahuja KDK, Watson G, O'Brien JA, Williams AD. Effects of Activity Tracker Use With Health Professional Support or Telephone Counseling on Maintenance of Physical Activity and Health Outcomes in Older Adults: Randomized Controlled Trial. *Jmir Mhealth and Uhealth.* 2021;9(1).

5. Cadmus-Bertram LA, Marcus BH, Patterson RE, Parker BA, Morey BL. Randomized Trial of a Fitbit-Based Physical Activity Intervention for Women. *American journal of preventive medicine.* 2015;49(3): 414-418.

6. Delbaere K, Valenzuela T, Lord SR, et al. E-health StandingTall balance exercise for fall prevention in older people: results of a two year randomised controlled trial. *Bmj-British Medical Journal.* 2021;373.

7. Van Dyck D, Herman K, Poppe L, Crombez G, De Bourdeaudhuij I, Gheysen F. Results of MyPlan 2.0 on Physical Activity in Older Belgian Adults: Randomized Controlled Trial. *Journal of Medical Internet Research.* 2019;21(10).

8. Kawaguchi K, Nakagomi A, Ide K, Kondo K. Effects of a Mobile App to Promote Social Participation on Older Adults: Randomized Controlled Trial. *Journal of Medical Internet Research.* 2024;26.

9. Li N, Wang N, Xu Y, et al. The impacts of a mHealth platform-enabled lifestyle-integrated multicomponent exercise program on reversing pre-frailty in community-dwelling older adults: A randomized controlled trial. *International Journal of Nursing Studies.* 2025;167.

10. Lyons EJ, Swartz MC, Lewis ZH, Martinez E, Jennings K. Feasibility and Acceptability of a Wearable Technology Physical Activity Intervention With Telephone Counseling for Mid-Aged and Older Adults: A Randomized Controlled Pilot Trial. *Jmir Mhealth and Uhealth.* 2017;5(3).

11. Pomkai N, Potharin D, Widyastari DA, et al. Effectiveness of an mHealth Application for Physical Activity Promotion Among Thai Older Adults: A Randomized Controlled Trial. *Inquiry-the Journal of Health Care Organization Provision and Financing.* 2024;61.

12. Recio-Rodriguez JI, Gonzalez-Sanchez S, Tamayo-Morales O, et al. Changes in lifestyles, cognitive impairment, quality of life and activity day living after combined use of smartphone and smartband technology: a randomized clinical trial (EVIDENT-Age study). *Bmc Geriatrics.* 2022;22(1).

13. Volders E, Bolman CAW, de Groot RHM, Verboon P, Lechner L. The Effect of Active Plus, a Computer-Tailored Physical Activity Intervention, on the Physical Activity of Older Adults with Chronic Illness(es)-A Cluster Randomized Controlled Trial. *Int J Environ Res Public Health.* 2020;17(7).

14. Zhou M, Zhang N, Zhang Y, et al. Effect of Mobile-Based Lifestyle Intervention on Weight Loss among the Overweight and Obese Elderly Population in China: A Randomized Controlled Trial. *International Journal of Environmental Research and Public Health.* 2021;18(16).
